# Prognostic impact of spatial niches in prostate cancer

**DOI:** 10.1038/s41598-026-35720-1

**Published:** 2026-01-17

**Authors:** Felix Schneider, Sarah Heike Böning, Beatriz Coelho Antunes, Adam Kaczorowski, Magdalena Görtz, Viktoria Schütz, Johannes Huber, Albrecht Stenzinger, Markus Hohenfellner, Stefan Duensing, Anette Duensing

**Affiliations:** 1 Molecular Urooncology, Department of Urology, University Hospital Heidelberg, Im Neuenheimer Feld 517, 69120 Heidelberg, Germany; 2 Department of Urology, University Hospital Heidelberg, and National Center for Tumor Diseases (NCT), Im Neuenheimer Feld 420, 69120 Heidelberg, Germany; 3 Institute of Pathology, University Hospital Heidelberg, Im Neuenheimer Feld 224, 69120 Heidelberg, Germany; 4 Precision Oncology of Urological Malignancies, Department of Urology, University Hospital Heidelberg, Im Neuenheimer Feld 517, 69120 Heidelberg, Germany

**Keywords:** Prostate cancer, Tumor heterogeneity, Spatial biology, Digital spatial profiling, Prognosis, Biomarker, Biomarkers, Cancer, Computational biology and bioinformatics, Oncology

## Abstract

The formation of intratumoral spatial niches has been reported for many human malignancies. However, the translational potential of such spatial niches is understudied. Herein, we utilize digital spatial profiling (DSP) to explore the prognostic relevance of spatially defined protein expression in high-risk prostate cancer. A total of 49 patient samples were analyzed for the expression of 46 proteins in 463 regions of interest (ROIs) from the tumor center (*n* = 198) and the tumor periphery (*n* = 265) resulting in 21,298 primary data points (mean per patient n = 9.4). Expression data from either the tumor center or the tumor periphery were not found to be prognostic. Protein expression of tumor center and periphery was then integrated into single datapoints by calculating the log_2_-transformed relative expression between the two niches for each protein and patient. Unsupervised hierarchical clustering of these data yielded two distinct patient subgroups. These clusters did not show a statistically significant correlation with known prognostic parameters yet significantly correlated with progression-free survival (*p* = 0.014, log-rank, HR 0.43; 95% CI, 0.22–0.86). Our results thus reveal that spatial protein expression contains prognostic information, however, only when expression data from both spatial niches are taken into account. In conclusion, our proof-of-concept study shows that DSP can be exploited for the development of novel prognostic biomarkers that rely on spatially resolved protein expression.

**Supplementary Information:**

The online version contains supplementary material available at 10.1038/s41598-026-35720-1.

## Introduction

Intratumoral heterogeneity is a characteristic feature of many human malignancies and involves the formation of spatial niches populated with tumor cells harboring certain functional properties. Such niches have been reported for urological malignancies, such as prostate and kidney cancer, but also for thyroid, lung and breast cancer^1–6^.

Prostate cancer is the most common non-cutaneous malignancy in men^7^. Although the majority of tumors have an indolent clinical course, a sizeable number of patients experience rapid progression and lethal disease outcome. Current risk stratification of prostate cancer patients mainly includes clinical and histopathological variables such as TNM stage, PSA level and Gleason score/grade group. Molecular risk factors for tumor progression include pathogenic mutations in DNA damage repair and checkpoint genes such as *BRCA2* or *TP53*^8,9^. Of note, the importance of intratumoral heterogeneity in prostate cancer has been acknowledged for decades and is incorporated into the prognostic Gleason score during histopathological grading. The Gleason score combines the most frequent and the most aggressive grade (when assessed in prostate biopsies) or the two most prevalent grades (when assessed in radical prostatectomy specimens).

Spatial biology has significantly improved the understanding of intratumoral heterogeneity and the formation of intratumoral niches^10^. This methodology capitalizes on the notion that a malignant tumor is not a mass of homogenous cells but rather a complex ‘ecosystem’, in which a heterogeneous population of tumor cells directly or indirectly interacts with a heterogeneous population of non-tumorous cells, thereby leading to bidirectional alterations of functional properties.

Several technical platforms to measure gene expression in a spatially defined manner have been developed^11^. One focus of most platforms lies in the use of archival formalin-fixed, paraffin embedded (FFPE) tissue samples, as these allow a correlation of spatial expression data with longitudinal clinical parameters, most importantly patient survival. Digital spatial profiling (DSP, Nanostring/Bruker®) of RNA and/or protein expression combines the power of high-plex approaches with the versatility of investigator-driven selection of regions of interest (ROIs) in FFPE tissue^12^. Whether spatially resolved gene expression data have the potential to be translated into improving clinical decision-making is still understudied.

In the present proof-of-concept study, we employed DSP on 49 prostate cancer specimens and developed a simple algorithm that incorporates information of spatial protein expression in the tumor center and periphery. Remarkably, only combining information from both spatial niches conveyed prognostic information. Our results underscore that DSP can be harnessed for the development of a novel class of prognostic biomarkers that is based on spatial information.

## Patients and methods

### Patients

A total of 49 prostate cancer patients with high-risk features (Table 1) were included in this proof-of-concept study. Tissue samples were obtained from radical prostatectomy specimens. All patients had undergone surgery at the Department of Urology of the University Hospital Heidelberg, Germany.

**Table 1:** Clinicopathological patient characteristics.

Patient characteristics (*n* = 49)	
Age, years, median (range)	63.3 (42–76)
PSA, µg/L, median (range)	19.0 (1.1–104.0)
Gleason score, *n* (%)	
7 (3 + 4)	4 (8.2)
7 (4 + 3)	17 (34.7)
8 (4 + 4)	7 (14.3)
9 (4 + 5)	17 (34.7)
9 (5 + 4)	3 (6.1)
10 (5 + 5)	1 (2.0)
Mutations, *n* (%)	
* BRCA1/2*	9 (18.4)
* TP53*	1 (2.0)
No mutation	17 (34.7)
Not tested	22 (44.9)
c/p/yTNM stage, *n* (%)	
T2	8 (16.3)
T3	38 (77.6)
T4	2 (4.1)
Tx	1 (2.0)
N0	25 (51.0)
N1	23 (46.9)
Nx	1 (2.0)
M0	39 (79.6)
M1	9 (18.4)
Mx	1 (2.0)
Death from cancer, *n* (%)	
Yes	17 (34.7)
No	32 (65.3)

FFPE tissue sections were obtained from the tissue bank of the National Center for Tumor Diseases (NCT) Heidelberg. This study is in accordance with the regulations of the tissue bank as well as under approval of the Ethics Committee of the Medical Faculty Heidelberg of the University of Heidelberg (votes S-864/2019, S-287/2022).

The somatic mutation status was characterized by panel next generation sequencing using either the TruSightTM Oncology 500 panel (Illumina, Cambridge, UK) as described previously ^9,19^or the Oncopanel v.2.8 (Eurofins Genomics, Konstanz, Germany).

### Digital spatial profiling

The GeoMx® DSP platform (Bruker Spatial Biology, Seattle, WA, USA; formerly NanoString Technologies) was used for the multiplex analysis of protein expression in FFPE tissue samples.

The workflow was carried out according to the manufacturer’s protocol and has previously been described ^6,13,20^. Briefly, tissue sections were deparaffinized and rehydrated using multiple wash steps (3 × 5 min xylene, 2 × 5 min each in 100% ethanol, 95% ethanol and ddH_2_O). For antigen retrieval, the slides were boiled in 1 × citrate buffer (pH 6.0) for 15 min using a pressure cooker, followed by washing in TBS-T. The slides were blocked for 1 h at room temperature (RT) in a humidified chamber using Buffer W (Bruker). Overnight incubation with the GeoMx® Immune Cell Profiling Core assay as well as the GeoMx® PI3K/AKT and MAPK signaling assays and the GeoMx® Cell Death assay was performed at 4 °C in a humidified chamber (Suppl. Table 1). To provide general tissue morphology, tissue sections were stained at the same time for pan-cytokeratin (epithelial cells) and CD45 (immune cells) using the Solid Tumor TME Morphology Kit (Bruker).

After antibody incubation, tissue sections were washed in TBS-T (3 × 10 min) and fixed with 4% paraformaldehyde for 30 min at RT. After washing the tissue sections in TBS-T (2 × 5 min), nuclei were stained using SYTO13 (Bruker) for 15 min at RT and washed again in TBS-T. Tissue sections were then either directly analyzed using the GeoMx® instrument or covered with Fluoromount-G® (Southern Biotech, Birmingham, AL, USA) and a cover slip and stored at 4 °C until used.

After slide scanning, ROIs were selected in the tumor center and periphery. After UV illumination of the ROIs, barcodes were collected and hybridized with fluorescent probes for 16 h at 67 °C. The hybridization products were purified by the nCounter® MAX/FLEX Prep Station (v4.1.0.1; Bruker) and counted using the nCounter® Digital Analyzer (v4.0.0.3; Bruker). Basic quality control was performed on the GeoMx® instrument, and data were normalized to Histone H3, S6 and GAPDH before further analyses were performed as described below.

### Statistical analysis and R packages

The R packages survival (v.3.5–5) and survminer (v.0.4.9) were used to generate Kaplan–Meier curves. A p-value of  ≤ 0.05 was considered statistically significant. Fisher’s exact test was used where indicated. For visualization and generation of the heatmaps, the R packages tidyverse (v.2.0.0), ggpubr (v.0.6.0) and ComplexHeatmap (v.2.14.0) were used. A Cox regression analysis was performed using R (v.4.2.0). Univariate analyses included the variables cluster, age, Gleason score, initial PSA value, TNM stage and mutation status to estimate hazard ratios, confidence intervals and p-values adjusted for multiple testing. For multivariate analyses, the events per variable rule limited models to three variables. Variables with *p* < 0.1 and independence as determined by the chi-square test were included. Several multivariate models were tested and the one with the lowest Akaike information criterion (AIC) value, which included cluster, initial PSA, and M stage, was selected for the final analysis. ANOVA confirmed this model’s superior fit.

## Results

### Tumor center and periphery are distinct functional niches but do not convey prognostic information on their own

**Fig. 1:**
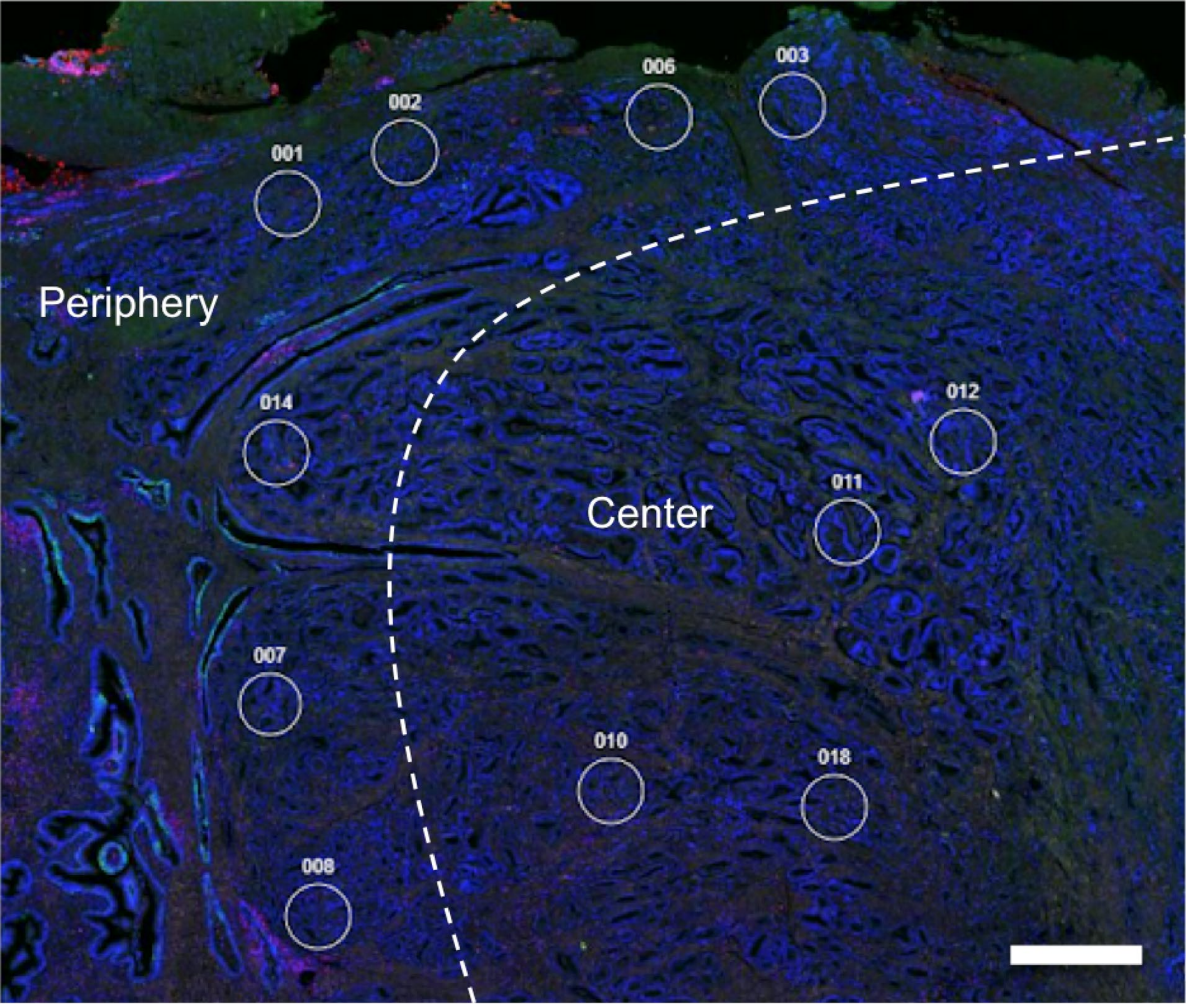
Digital spatial profiling (DSP) of spatial niches in prostate cancer. Representative DSP analysis to evaluate protein expression in prostate cancer. Regions of interest (ROIs) representing the tumor center and periphery are shown as white circles. Tissue was stained with anti-PanCK (green), anti-CD45 (pink) and SYTO13 (blue, nuclear staining) for morphological visualization. Scale bar = 1 mm.

A total of 49 high-risk prostate cancer samples were analyzed by DSP for the expression of 46 proteins (Suppl. Table 1). Regions of interest (ROIs; *n* = 463) from the tumor center (*n* = 198) and the tumor periphery (*n* = 265) were selected, resulting in a total of 21,298 primary data points (mean per patient n = 9.4). The tumor periphery was defined as being directly adjacent to non-malignant prostate tissue. ROIs in the tumor center only included viable tumor cells and no visible areas of necrosis. An example of the ROI selection is shown in Fig. 1.

**Fig. 2:**
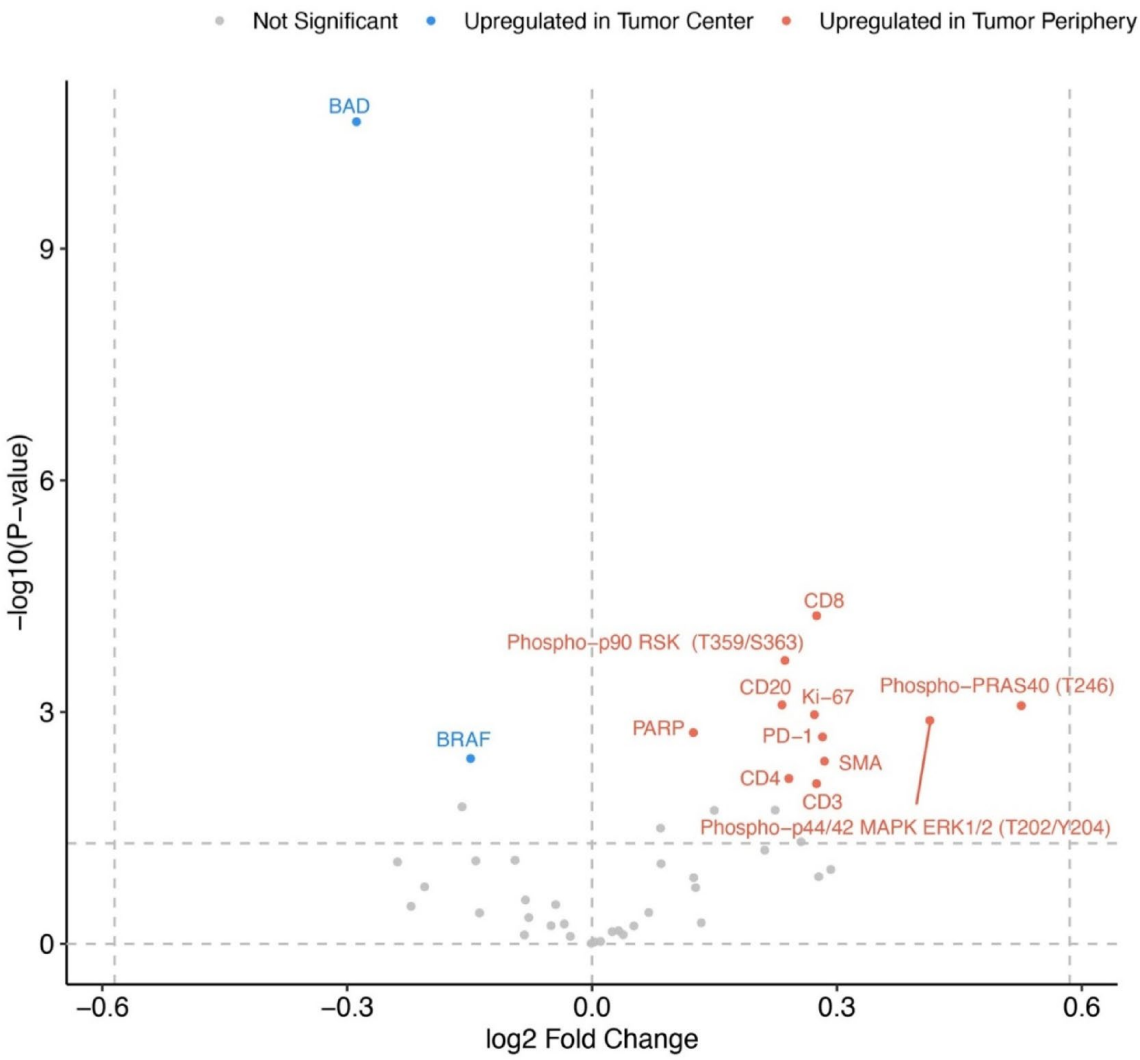
Differentially expressed proteins in tumor center and tumor periphery. Data points are colored by significance, adjusted for multiple testing using the Benjamini–Hochberg procedure (FDR). *P*-values were derived from linear mixed-effects models (LMM) assessing the effect of region (periphery *vs.* center). The horizontal line marks the p-value cutoff at 0.05.

The mean expression of each protein was calculated for the tumor center and tumor periphery of each sample, giving rise to 2,254 data points for each area. To visualize significantly up- or downregulated proteins in the tumor center versus the tumor periphery, a volcano plot was generated (Fig. 2). Most differentially expressed proteins showed an upregulation in the tumor periphery, a distinctive feature of our cohort. Of note, the most significantly upregulated protein in the tumor center was BAD, which is in line with a previous report from our group^13^.

**Fig. 3:**
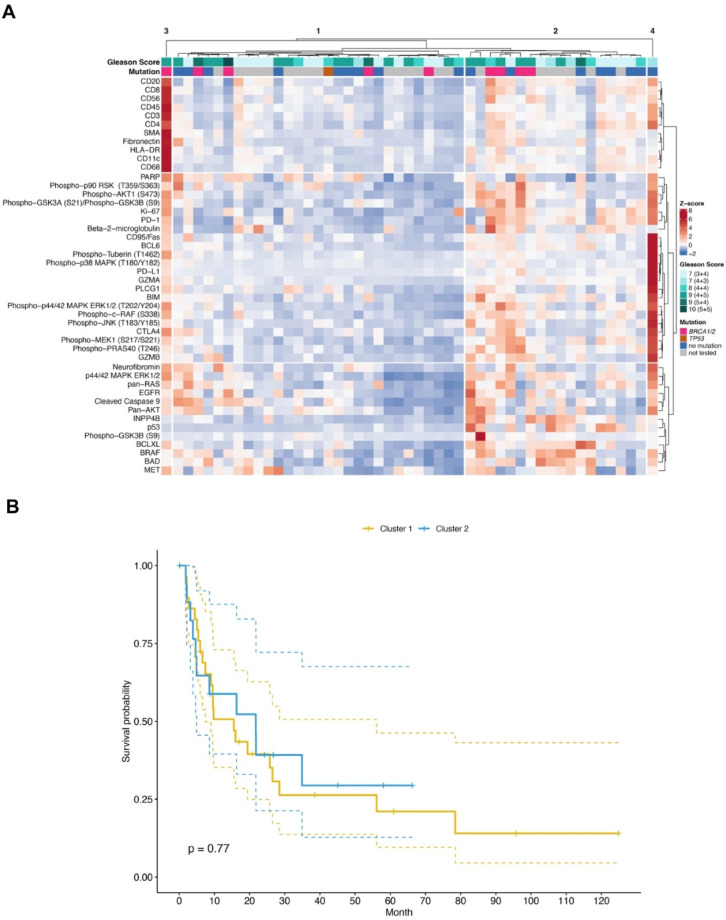
DSP of protein expression in the tumor center is not prognostic. (**A**) Unsupervised clustering and heatmap of DSP protein expression data obtained from ROIs of the tumor center. For clustering, protein expression values were z-transformed by subtracting the mean protein expression from each protein expression value and dividing it by its standard deviation. Z-scores are presented according to the color key. Two patients, representing clusters 3 and 4, respectively, were considered outliers and not included in further analyses. Gleason scores and the presence of pathogenic mutations in *BRCA1*/*BRCA2* or *TP53* are shown per patient (grey = not tested). (**B**) Kaplan–Meier curve showing progression-free survival (PFS) of 47 patients in cluster 1 and cluster 2. Log-rank statistics is shown.

**Fig. 4:**
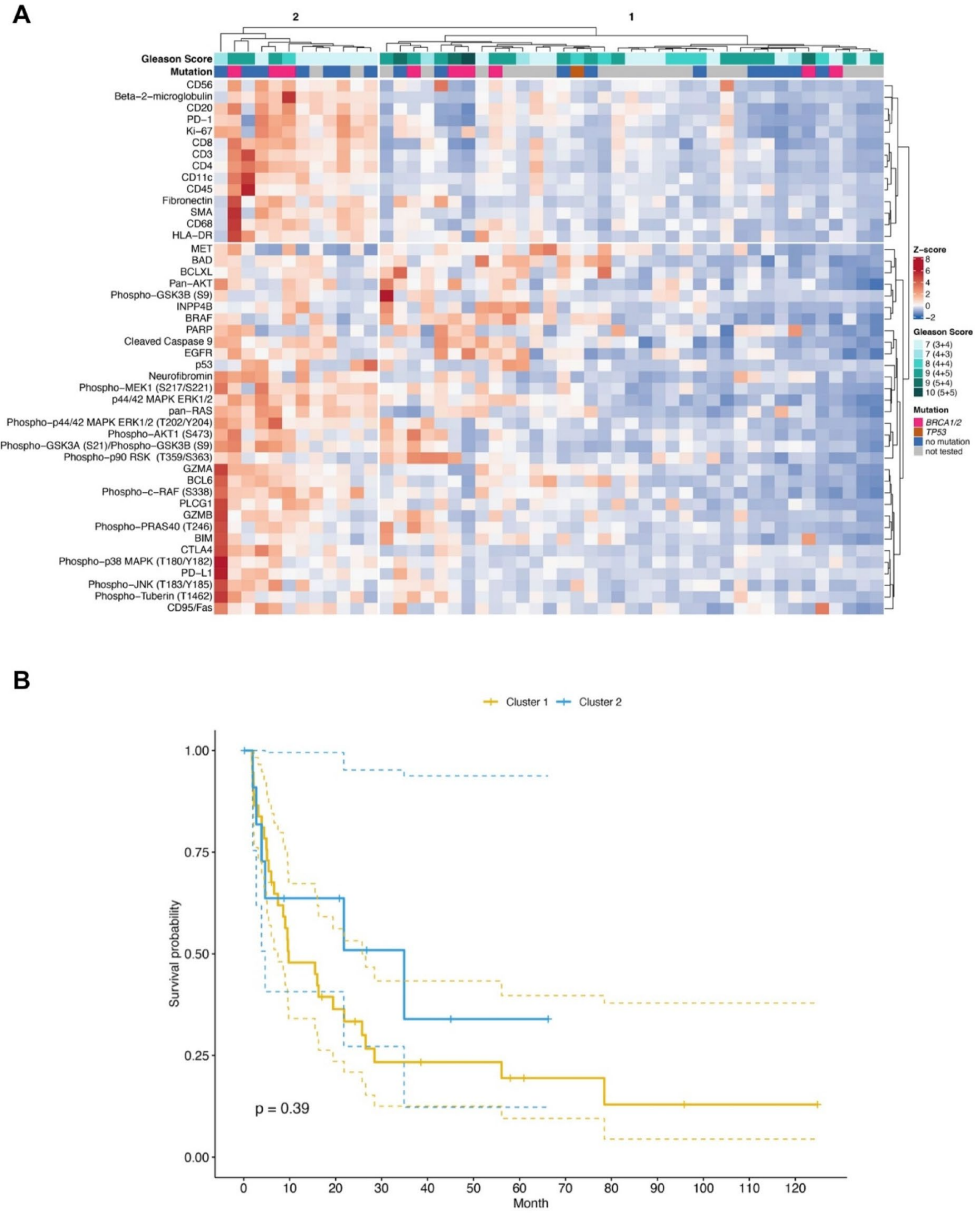
DSP of protein expression in the tumor periphery is not prognostic. (**A**) Unsupervised clustering and heatmap of DSP protein expression data obtained from ROIs of the tumor periphery. For clustering, protein expression values were z-transformed by subtracting the mean protein expression from each protein expression value and dividing it by its standard deviation. Z-scores are presented according to the color key. Gleason scores and the presence of pathogenic mutations in *BRCA1*/*BRCA2* or *TP53* are shown per patient (grey = not tested). (**B**) Kaplan–Meier curve showing PFS of 49 patients in cluster 1 and cluster 2. Log-rank statistics is shown.

To further analyze protein expression in the tumor center and periphery, unsupervised hierarchical cluster analyses were performed for each spatial compartment (Figs. 3, 4). Two major patient clusters were identified for each niche (Figs. 3A, 4A). Neither cluster correlated with known prognostic parameters, such as Gleason score or mutational status of *BRCA1/2* or *TP53*. To test whether protein expression in the tumor center or tumor periphery conferred prognostic information, clusters of each compartment were correlated with progression-free survival (PFS; Figs. 3B, 4B). However, there was no statistically significant correlation between patient clusters and PFS in either compartment (tumor center, *p* = 0.77; tumor periphery, *p* = 0.39, log-rank statistics; Figs. 3, 4).

### Integration of spatial information identifies protein expression clusters that carry prognostic significance

We then asked whether integrating the spatial information of tumor center and periphery would be more informative with respect to patient outcome. The mean expression of each protein was calculated for tumor center and tumor periphery, and the log_2_-transformed relative expression of each protein was determined for each patient [Eq. (1)].

$$relative\,expression = {\mathrm{log}}_{2} \frac{{mean \left( {expression\,tumor\,periphery} \right)}}{{mean \left( {expression\,tumor\,center} \right)}}$$

**Fig. 5:**
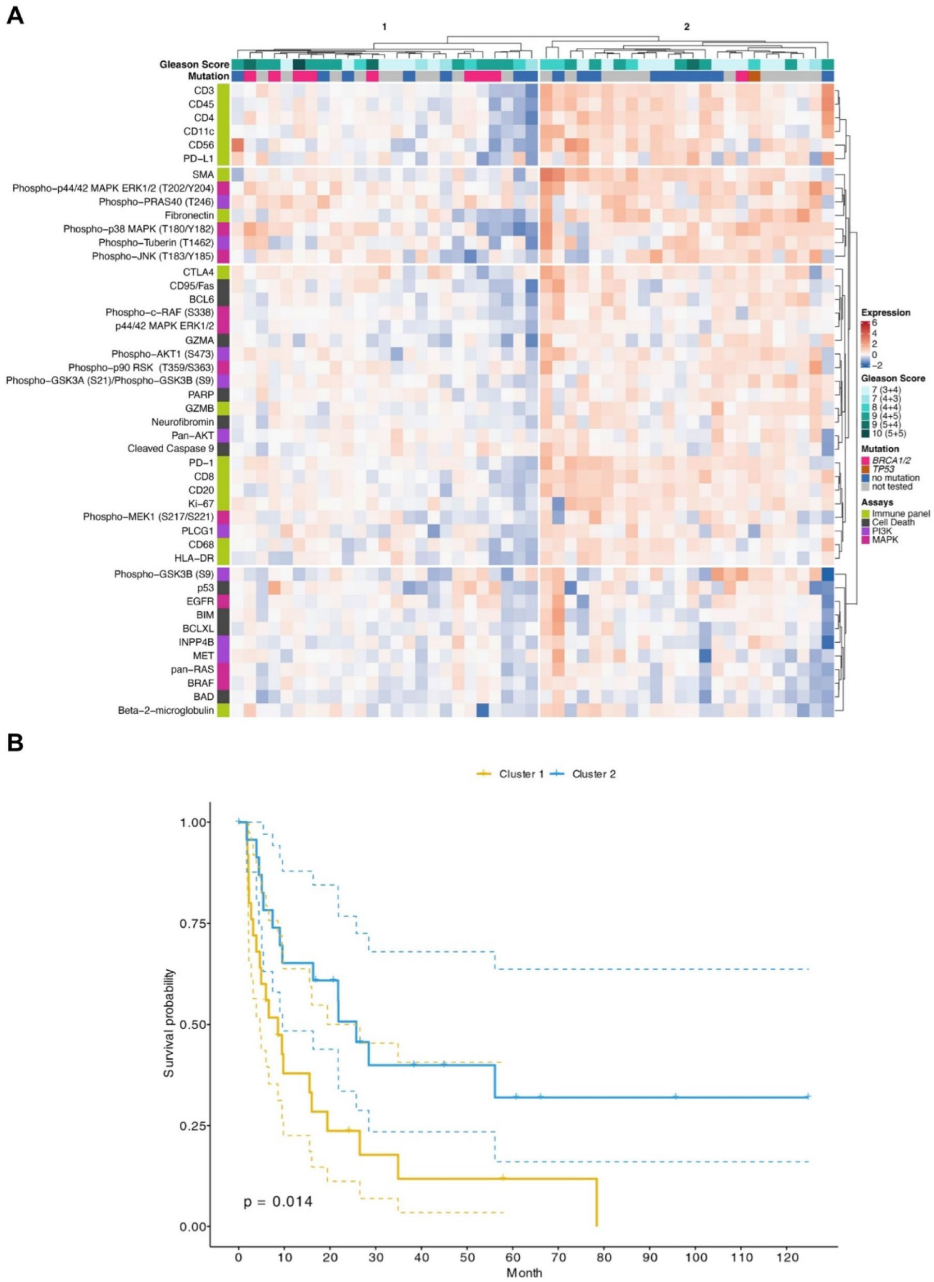
Integration of DSP results from tumor periphery and tumor center conveys prognostic information. (**A**) Unsupervised clustering and heatmap of integrated DSP protein expression data [Eq. (1)]. Gleason scores and the presence of pathogenic mutations in *BRCA1*/*BRCA2* or *TP53* are shown per patient (grey = not tested). Proteins are colored by the assay panel each protein belongs to (Immune panel, Cell Death, PI3K, MAPK). (**B**) Kaplan–Meier curve showing PFS of 49 patients in cluster 1 and cluster 2. Log-rank statistics is shown.

Applying this equation, positive relative expression values indicate a higher expression of the respective protein in the tumor periphery whereas negative relative expression values indicate a higher expression in the tumor center. This allowed each protein to be represented by a single data point without the loss of spatial information. An unsupervised hierarchical cluster analysis of the results showed a robust separation of the 49 patients into two relative expression clusters (Fig. 5A).

A Kaplan–Meier analysis to test for differences in survival between the two patient clusters (Fig. 5B) showed that patients in cluster 2 displayed a significantly better PFS when compared to patients in cluster 1, with a median PFS of 25.7 months after radical prostatectomy compared to a median PFS of 8.6 months in cluster 1 (*p* = 0.014). A univariate Cox regression analysis showed that cluster association (hazard ratio, HR, 2.3, confidence interval, CI, 1.2–4.6, *p* = 0.016), initial PSA value (HR, 2.4, CI, 1.2–4.9; *p* = 0.017) and M stage (HR, 5.0, CI, 2.1–12, *p* < 0.001) correlated significantly with the risk for progression. A multivariate model selected for the best balance of fit and complexity retained all three variables, and cluster association showed a trend towards statistical significance (HR, 1.9, CI, 0.95–3.9; *p* = 0.069). Of note, the initial PSA value did not retain statistical significance (HR, 1.7, CI, 0.79–3.7; *p* = 0.171), while M stage remained significant (HR, 3.8, CI, 1.55–9.3; *p* = 0.004).

To assess the effect sizes of patient clusters *versus* individual proteins, we calculated their respective HR and p-values. While several proteins were associated with risk of progression (PD-1, CD56, CD8, CD4, CD20, CD3, phospho-GSK3B S9; Suppl. Table 2), the effect of the clusters (HR 2.3; *p* = 0.016) was larger than the effect of any single protein. We then tested whether the cluster effect is driven by one or a few very strong proteins rather than the combined spatial pattern using a multivariate Cox model. To avoid overfitting, the top three most significant proteins were included in addition to the cluster. Cluster 1 remained significantly associated with a higher risk of progression (HR 2.2, *p* = 0.036), while none of the individual proteins were significant (Suppl. Table 3), indicating that the cluster’s prognostic value is not driven by any single protein.

**Fig. 6:**
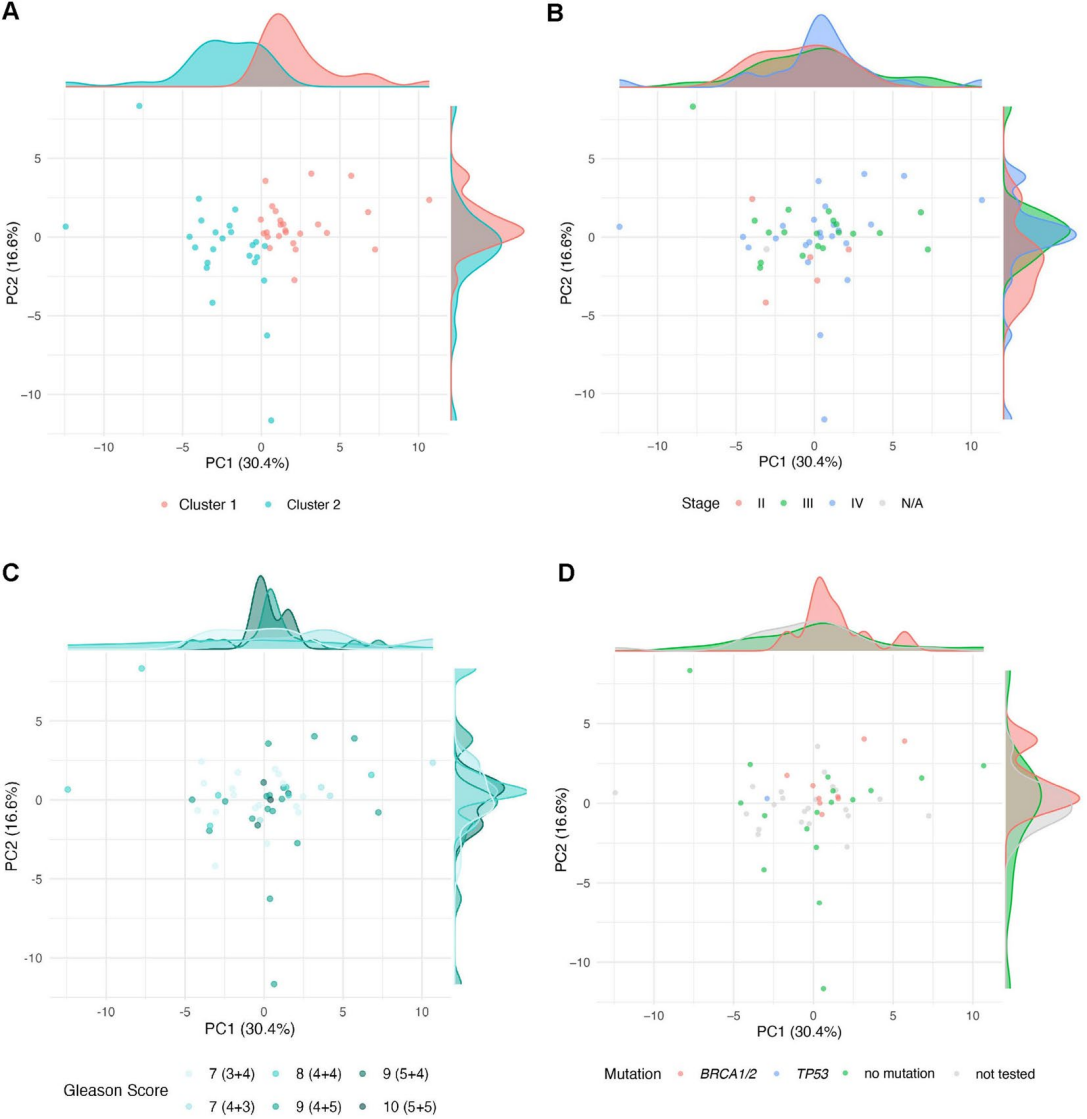
Principal component analysis (PCA) of all patients (*n* = 49) based on the mean expression of 46 proteins between tumor center and periphery. Each point represents an individual patient. Color coding according to the following variables: (**A**) cluster, (**B**) stage, (**C**) Gleason score and (**D**) mutational status.

There was no statistically significant correlation between the two patient clusters and known clinical parameters, such as Gleason score (*p* = 0.39; Fisher’s exact test) or the presence of pathogenic mutations in *BRCA1/2* or *TP53*. Although there was a trend towards enrichment of mutational events in patient cluster 1 (Fig. 5A), it did not reach statistical significance (*p* = 0.11; Fisher’s exact test). Principal component analyses confirmed the separation of the two expression clusters (Fig. 6A) as well as the lack of correlation with stage, Gleason score, mutational status (Fig. 6B–D) or age (SFig. 1).

**Fig. 7:**
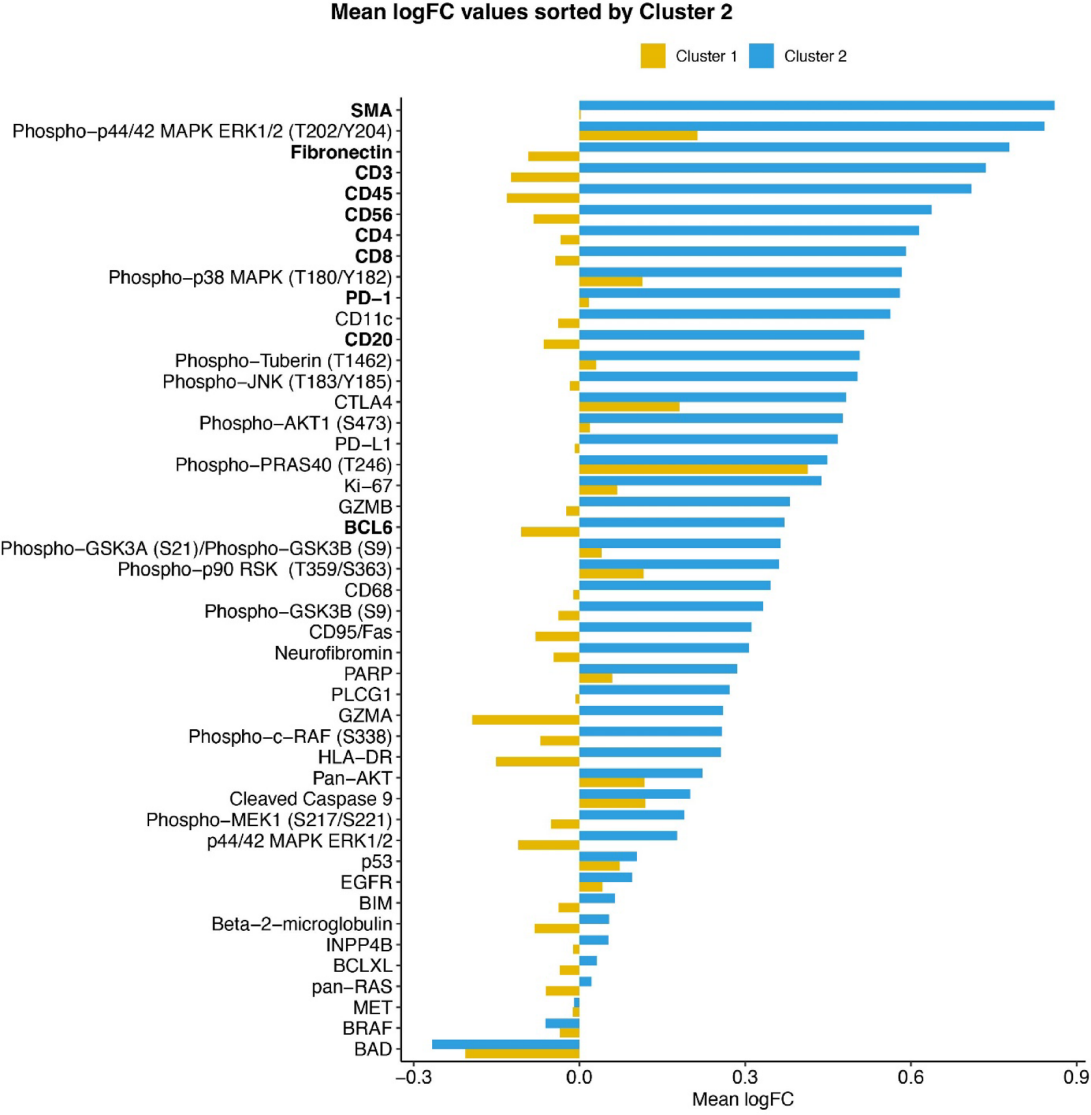
Differential protein expression according to clusters. Mean log fold-change (logFC) for each protein and cluster, sorted according to the values from cluster 2. The top ten differentially expressed proteins are labeled in bold.

With respect to protein expression, patient cluster 2 was found to be enriched for samples showing an upregulation of proteins in the tumor periphery (Figs. 5A, 7). These proteins were clustered into distinct functional groups (Fig. 5A). For example, protein cluster 1 was entirely comprised of proteins involved in immune regulation, whereas protein cluster 2 mostly contained phosphorylated (activated) signaling proteins of the PI3K and MAPK pathways. Proteins that were downregulated in patient cluster 2 also grouped together (protein cluster 4). This cluster was characterized by proteins involved in apoptosis regulation and cell signaling.

The top ten differentially expressed proteins in patient clusters 1 and 2 showed an upregulation in the tumor periphery and included CD3, CD20, CD8, CD45, PD-1, fibronectin, SMA, CD56, CD4 and BCL6 (Table 2; Figs. 5A, 7). Interestingly, these proteins fall into two main functional categories that are either expressed by immune cells or involved in epithelial-to-mesenchymal transition (EMT). Of note, eight of these proteins (exception: CD20, BCL6) belong to the top ten overexpressed proteins of cluster 2 (mean logFC; Fig. 7, labeled in bold).

**Table 2:** Top ten differentially expressed proteins.

Protein	*p*-value*	FDR	Mean logFC cluster 1	Mean logFC cluster 2	Difference
CD3	3.00E-09	1.70E-07	-0.124	0.735	0.859
CD20	3.30E-08	5.90E-07	-0.064	0.516	0.578
CD8	3.90E-08	5.90E-07	-0.044	0.591	0.635
CD45	5.11E-07	5.88E-06	-0.131	0.709	0.841
PD-1	1.71E-06	1.57E-05	0.017	0.580	0.563
Fibronectin	5.21E-06	3.42E-05	-0.093	0.778	0.871
SMA	5.21E-06	3.42E-05	0.002	0.860	0.858
CD56	8.33E-06	4.79E-05	-0.083	0.637	0.720
CD4	1.83E-05	9.34E-05	-0.034	0.615	0.649
BCL6	8.56E-05	3.94E-04	-0.105	0.371	0.476

*Mann–Whitney U test with false discovery rate (FDR) correction.

Collectively, our results demonstrate that spatial information of protein expression can be harnessed for the development of novel prognostic tools in prostate cancer.

## Discussion

Spatial niches have been reported in a number of human malignancies, where they contribute to intratumoral heterogeneity^1–6^. However, the prognostic impact of such niches is largely unknown.

In the present proof-of-concept study, we show that the ratio of protein expression between tumor periphery and tumor center in high-risk prostate cancer contains prognostic information. Unsupervised hierarchical cluster analysis revealed a robust separation of patients into two expression clusters with statistically significant differences in PFS. Importantly, only the combination of spatial information from tumor periphery and tumor center was prognostic while data from each niche when analyzed separately were not.

Several previous studies have used spatially defined gene expression analysis in prostate cancer. An early report using spatial transcriptomics showed an upregulation of immune-related genes in the tumor periphery, which is in line with findings presented here, although a correlation with patient survival was not performed in the earlier study^14^. Other reports explored differences between the tumor and the adjacent tumor stroma. A study focusing on single cell and spatial transcriptomics showed an immunosuppressive tumor microenvironment associated with myeloid cell populations and exhausted T as well as NK cells but activated B cells^15^. Interestingly, these alterations were not only detected in tumor samples but also in samples from normal tissue adjacent to the tumor when compared to healthy tissue. Another study used DSP to characterize the tumor and adjacent stroma and identified differentially expressed immune markers (OXL40L, CTLA4, CD11c), which conveyed prognostic information^16^. Results presented in our current study suggest the presence of an intratumoral gradient of immune cell populations with the tumor center being immunologically distinct from the tumor periphery. Other studies focus on differences between the primary tumor and metastatic lesions. Brady et al. used DSP to characterize metastatic prostate cancer and found that most metastases lacked immune cell infiltrates and showed a high expression of immune checkpoint proteins, such as B7-H3/CD276 and TIM3^17^. Another study showed that primary tumors from castration-resistant prostate cancer patients contain T cells that may be suppressed by neighboring regulatory T cells^18^.

All reports above used spatial gene expression analysis to better characterize the immune landscape of prostate cancer. However, it is worth mentioning that non-immunological functional spatial niches also exist and have been described in prostate cancer. For example, an earlier study from our group demonstrated that the tumor center of prostate cancer specimens is characterized by a high expression of phosphorylated BAD, a member of the BCL2 family of proteins that regulate apoptosis^13^. Results shown in this current report confirm our previous observation.

Limitations of the present proof-of-concept study are the relatively small patient cohort, its retrospective nature and that it was carried out at a single institution. The fact that pre-defined protein panels were used for our analysis limited the number of proteins and target categories that were analyzed. The DSP platform is cost-intensive and not widely available. Hence, future validation of our results by conventional immunohistochemistry will be crucial to translate our findings into clinical practice.

In addition, we would like to point out that the prognostic impact of patient clusters in the multivariate analysis did not reach statistical significance. In contrast to the M stage, which retained statistical significance, only a statistical trend was detected for the two patient clusters. We explain this finding by the relatively small cohort size and the fact that most patients showed high-risk features. This relatively uniform cohort composition was compromised by patients with synchronous metastasis (almost 20%), which very likely added cluster heterogeneity and thereby subverted the statistical power. Future studies will have to address this problem by expanding the number of patients and focusing on a more homogeneous patient population.

## Conclusion

Our results add spatially resolved protein expression in the tumor center and periphery to the emerging class of prognostic oncologic biomarkers that rely on spatial information.

## Supplementary Information

Below is the link to the electronic supplementary material.

Supplementary Material 1

## Data Availability

The data is provided within the manuscript or supplementary information files.
