# Supplementary material for: Prognostic impact of spatial niches in prostate cancer
Source: Sci Rep. 2026 Jan 17;16:2598. doi: 10.1038/s41598-026-35720-1 (PMC12820118; doi:10.1038/s41598-026-35720-1)
Supplement: Supplementary file 1 — Supplementary Material 1 [file 41598_2026_35720_MOESM1_ESM.docx]

**SUPPLEMENTARY INFORMATION**

**Prognostic impact of spatial niches in prostate cancer**

Felix Schneider, Sarah Heike Böning, Beatriz Coelho Antunes,

Adam Kaczorowski, Magdalena Görtz, Viktoria Schütz, Johannes Huber,

Albrecht Stenzinger, Markus Hohenfellner, Stefan Duensing, Anette Duensing

**SUPPLEMENTARY FIGURE**

**Supplementary Figure 1.** Principal component analysis (PCA) of all patients (n=49) based on the mean expression of 46 proteins between tumor center and periphery. Each point represents an individual patient. Color coding according to age group.


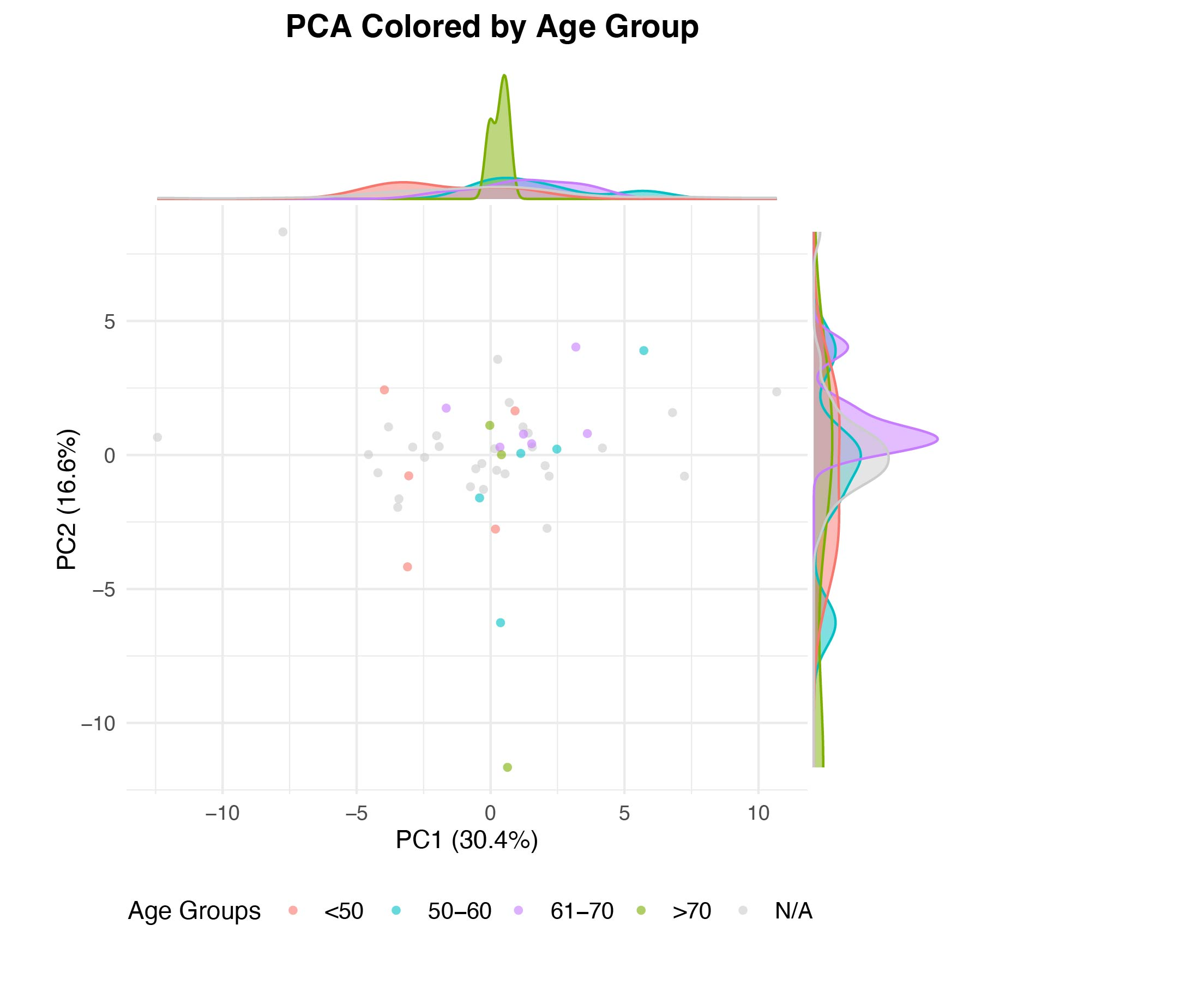


**SUPPLEMENTARY TABLES**

**Supplementary Table 1.** Overview of DSP targets.

| Assays | Targets |
| --- | --- |
| PI3K/AKT | Phospho-AKT1 (S473)  Pan-AKT  Phospho-GSK3B (S9)  Phospho-GSK3A (S21)/Phospho-GSK3B (S9)  INPP4B  MET  PLCG1  Phospho-PRAS40 (T246)  Phospho-Tuberin (T1462) |
| MAPK | BRAF  EGFR  Phospho-JNK (T183/Y185)  Phospho-p38 MAPK (T180/Y182)  Phospho-p44/42 MAPK ERK1/2 (T202/Y204)  p44/42 MAPK ERK1/2  Phospho-MEK1 (S217/S221)  Phospho-c-RAF (S338)  pan-RAS  Phospho-p90 RSK (T359/S363) |
| Cell Death | BAD  BCL6  BCLXL  BIM  CD95/Fas  Cleaved Caspase 9  GZMA  Neurofibromin  P53  PARP |
| Immune Cell Profiling | Beta-2-microglobulin  CD3  CD4  CD8  CD11c  CD20  CD45  CD56  CD68  PanCK  CTLA4  Fibronectin  GZMB  HLA-DR  Ki-67  PD-1  PD-L1  SMA |
| Controls | IgG (rabbit)  IgG1 (mouse)  IgG2a (mouse)  Histone H3  GAPDH  S6 |

**Supplementary Table 2.** Several proteins have individual associations with risk of progression.

| Cluster/Protein | Hazard Ratio (HR) | p-value |
| --- | --- | --- |
| PD-1 | 0.3 | 0.004 |
| CD56 | 0.4 | 0.007 |
| CD8 | 0.4 | 0.009 |
| CD4 | 0.4 | 0.020 |
| CD20 | 0.4 | 0.021 |
| CD3 | 0.5 | 0.031 |
| pGSK3B (S9) | 0.5 | 0.033 |

**Supplementary Table 3.** The clusters’ prognostic value is not driven by any single protein. Multivariate Cox model testing Cluster 1 against the top three significant proteins (see Suppl. Table 2) to avoid overfitting.

| Variable | Hazard Ratio (HR) | p-value |
| --- | --- | --- |
| Cluster 1 | 2.2 | 0.036 |
| PD-1 (low) | 1.2 | 0.727 |
| CD56 (low) | 1.7 | 0.359 |
| CD8 (low) | 1.8 | 0.205 |
